# A Valuable Aid to Diphtheria Control?

**Published:** 1920-02-07

**Authors:** 


### THE SCHICK REACTION.

### A Valuable Aid to Diphtheria Control?

In spite of the remarkable and successful results which have followed the widespread use of diphtheria antitoxin, there is in this country a curious reluctance to adopt certain measures in control of the disease, which have already been extensively employed in other lands, notably in America. For this reason, a paper recently published in the *Lancet* by Dr. H. Mason Leete should receive all the attention it deserves. This author describes, and discusses primarily, the Schick reaction for the determination of individual susceptibility to diphtheria, and although he does not make any point which is essentially new to the literature on the subject, yet his clearly arranged words may well serve to light the flame of enthusiasm which is at present wanting. Many similar laboratory-controlled procedures have been handicapped by non-availability for general and institutional purposes of the required biological products; but this cannot, in this instance, be the explanation, since we can affirm that at least one large producing company is prepared to supply them in graduated and considerable quantities, and is anxious to bring before both the medical profession and the local health authorities the possibilities of the method.

Admitting that most cutaneous reactions are far from infallible and that this one must be recommended with certain reserve as mentioned later, yet in view of the prevailing lack of precise knowledge on the subject, we find excuse for outlining the essentials of the Schick test as actually employed. Many articles, statistics, and temporary conclusions are found in the current medical journals of the U.S.A., but in that country the observational work is already so far advanced that they are not always easy for the uninitiated to follow.

To begin then at the beginning, the Schick test is carried out by injecting a very small dose of diphtheria toxin into the skin. This injection should be *intra*-cutaneous and of accurate quantity, which is in practice about one-fiftieth of a minimal lethal dose. The best site is the flexor aspect of the forearm, just below the fold of the elbow, and a round white wheal about the size of a large split pea should be formed. We may quote from Dr. Leete's own experience as to the typical positive reaction, but for variations, discrepancies, and difficulties we must refer the reader back to the author's article. "A typical positive reaction begins to show distinctly in from 24 to 48 hours, and reaches its height about the third day. It is a sharply circumscribed area of redness, with definite, though slight, infiltration, circular or somewhat oval in shape, and varying from half to one inch in diameter. This persists for about a week, and on fading leaves a brownish pigmented area which shows traces of desquamation. A negative reaction is shown by the absence of redness and infiltration; after 24 to 48 hours nothing can be seen, except a point of redness marking the needle track."

It is beyond the scope of this note even to outline the many experiments by which either the American physicians or Dr. Leete have arrived at their favourable conclusions, but after perusal of both, we feel justified in stating that the balance of evidence suggests the test to be of very definite value in detecting diphtheria susceptibles, and has, accordingly, many applications. Certainly it is very easy to perform and apparently free from any danger. Also, it would, although dependent upon the presence of corresponding antibodies in the blood, appear to be much more reliable than the known tuberculin reactions. If general experience runs parallel with that of Dr. Leete in finding an error of not more than 1 to 2 per cent., then its value is amply confirmed, but here it must be noted that a large portion of the evidence is obtained from people passively immunised with diphtheria antitoxin.

What we must with relative certainty know is whether the Schick test can be relied upon to the same extent to indicate the presence, or absence, of natural antitoxin and a consequent natural immunity. Dr. Leete's later experiences, and to a greater extent the American statistics, go a considerable distance towards proving that the wider application is justified, and, if so, the possible benefits which may result are manifold.

Diphtheria as a disease works its most deadly and most frequent ill upon young children, particularly upon those recently suffering from catarrhal and inflammatory conditions of the mucous membranes of nose and throat. Typically we see this in scarlet fever and measles convalescents. To be able to detect at least those children which are the more susceptible to such secondary diphtheric infection would be valuable for the patients and attendants alike, and opens up the possibility of guided production of protective passive immunity.

So much for the outlook in hospital and institutional practice and the question arises as to how far the same process can be applied to the general public. Except for that section which comes under the direct control of the local health authorities, although the indications would be identical, the practice of turning them to advantage would not be so easy. Through the agency of the school medical service the second most valuable possibility arises and in this connection it must be pointed out that for some time past the New York Board of Health has, when possible, applied the test to school children, with results known to be most favourable.

Therefore would we urge that more practical and experimental attention be given in this country to the Schick reaction; since, valuable though limited local observations are, it is only by extended trial over a considerable section of population that a satisfactory conclusion can be drawn. Possibly the subject might with advantage be taken up by the Medical Research Committee.

